# Affective temperaments mediate the effect of childhood maltreatment on bipolar depression severity

**DOI:** 10.1002/pcn5.94

**Published:** 2023-05-10

**Authors:** Itsuki Terao, Chihiro Morishita, Yu Tamada, Jiro Masuya, Yota Fujimura, Hiroyuki Toda, Ichiro Kusumi, Hajime Tanabe, Takeshi Inoue

**Affiliations:** ^1^ Department of Psychiatry Tokyo Medical University Tokyo Japan; ^2^ Department of Psychiatry Tokyo Medical University Hachioji Medical Center Tokyo Japan; ^3^ Department of Psychiatry National Defense Medical College Saitama Japan; ^4^ Department of Psychiatry Hokkaido University Graduate School of Medicine Hokkaido Japan; ^5^ Department of Social and Human Studies Faculty of Humanities and Social Sciences Shizuoka Japan

**Keywords:** affective temperament, bipolar disorder, childhood maltreatment, depressive symptom, Temperament Evaluation of Memphis, Pisa, Paris, and San Diego Autoquestionnaire (TEMPS‐A)

## Abstract

**Aim:**

Bipolar disorder is a leading disorder contributing to global disease burden, and bipolar depression often becomes severe and refractory. Therefore, clarifying the pathophysiology of bipolar disorder is an urgent issue. Previous reports suggested that factors, such as affective temperaments and childhood maltreatment, aggravate bipolar depression severity. However, to our knowledge, no reports to date have clarified the interrelationship between the above factors and bipolar depression severity. We here hypothesized that childhood maltreatment worsens bipolar depression severity via increasing affective temperaments. To test this hypothesis, a covariance structural analysis was conducted.

**Methods:**

The following information was evaluated for a total of 75 people with bipolar disorder using self‐administered questionnaires: demographic characteristics, depressive symptoms (Patient Health Questionnaire‐9), history of childhood maltreatment (Child Abuse and Trauma Scale), and affective temperaments (Temperament Evaluation of Memphis, Pisa, Paris, and San Diego Autoquestionnaire). The results were analyzed using covariance structure analysis.

**Results:**

A significant indirect effect of childhood maltreatment on bipolar depression severity via increasing affective temperaments was identified, whereas the direct effect of childhood maltreatment was not significant.

**Conclusion:**

Our results reveal that affective temperaments can mediate the adverse effects of childhood maltreatment on the severity of bipolar depression.

## Graphical abstract

Affective temperaments can mediate the association between childhood maltreatment and bipolar depression severity. This is the first study to identify the mediation effect of affective temperaments between childhood maltreatment and bipolar depression severity using covariance structure analysis.

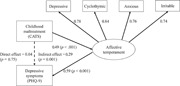

## INTRODUCTION

Bipolar disorder has been reported to affect 39 million people worldwide, and the disability‐adjusted life year has been estimated to be about 8.59 million years.^1^ A previous study indicated that the rate of suicide in people with bipolar disorder is four times higher than that in the general population.^2^ Therefore, elucidating the pathophysiology of bipolar depression is crucial.

In a review of twin studies, the heritability of bipolar disorder was reported to be approximately 60%–85%, suggesting that genetic factors contribute substantially to its pathogenesis. However, as heritability is not 100%, this suggests that environmental factors also contribute to the remaining 15%–40% of cases.^3^ Among the possible environmental factors that affect the onset and course of bipolar disorder, many studies have indicated that childhood maltreatment may be involved.^4^, ^5^, ^6^, ^7^

The onset of bipolar disorder is most common at about the age of 19 years, which may be a long time after a patient has experienced childhood maltreatment.^8^ Therefore, it is assumed that there are mediators between childhood maltreatment and the onset and course of bipolar disorder. Lippard and Nemeroff^9^ reported the mediation effects of inflammation, immune system dysfunction, changes in the hypothalamic–pituitary–adrenal axis, and epigenetics between childhood maltreatment and mood symptoms in adults with mood disorders. We have focused on the possible mediating effects of affective temperaments between childhood maltreatment and the diagnosis and severity of mental disorders in adulthood.^10^, ^11^, ^12^, ^13^ Affective temperaments were originally described by Kraepelin as basic states of manic–depressive illness, and its definition was subsequently modified by von Zerssen and Akiskal as comprising depressive, cyclothymic, irritable, anxious, and hyperthymic temperaments.^14^ We previously demonstrated using covariance structural analysis that childhood maltreatment is significantly positively associated with increased affective temperaments (i.e., cyclothymic, depressive, irritable, and anxious temperaments); we also showed that affective temperaments act as a mediator between childhood maltreatment and the diagnoses of major depressive disorder and bipolar disorder, and that they increase depression severity in the general population and in people with major depressive disorder.^10^, ^11^, ^12^, ^13^

Although a significant correlation between affective temperaments and bipolar depression severity was reported in a cross‐sectional study by Luciano et al.,^15^ the association between childhood maltreatment and affective temperaments and the mediating effect of affective temperaments on childhood maltreatment and adult depression severity in people with bipolar disorder have not yet been reported. Therefore, in this study, we hypothesized that childhood maltreatment increases affective temperaments, and increases bipolar depression severity via increasing affective temperaments, and tested this hypothesis in 75 people with bipolar disorder using covariance structure analysis.

## METHODS

### Subjects

A total of 75 outpatients with bipolar disorder who were being treated at Hokkaido University Hospital, National Defense Medical College Hospital, Japan Self Defense Forces Sapporo Hospital, and Self‐Defense Forces Central Hospital were recruited between April 2012 and April 2013. All subjects were evaluated and diagnosed by psychiatrists with more than 5 years of experience in psychiatric practice, and who were proficient in treating bipolar disorder. The inclusion criteria were as follows: (1) a primary diagnosis of bipolar disorder according to *DSM‐Ⅳ‐TR*; (2) being 20 years of age or older; and (3) having the ability to agree to the study. The following were the exclusion criteria: (1) not having an uncontrolled or serious medical condition; (2) having a diagnosis of Axis‐II personality disorder according to the *DSM‐Ⅳ‐TR*; and (3) having a present or history of diagnosis of an organic disorder causing psychiatric symptoms.

All the subjects provided written informed consent when they entered the study. This study was conducted according to the 1964 Declaration of Helsinki, as revised in 2008. In addition, approval was obtained from the institutional review boards of National Defense Medical College (study approval number: 4284), Hokkaido University Hospital (009‐0143), and Tokyo Medical University (SH4098).

### Questionnaires

#### Patient Health Questionnaire‐9

The Patient Health Questionnaire‐9 (PHQ‐9) is a self‐administered questionnaire that evaluates the severity of depressive symptoms.^16^ The high reliability and validity of the Japanese version have been verified.^17^ The PHQ‐9 consists of nine questions, each rated on a 4‐point Likert scale, and a higher total score is interpreted as indicating severer depressive symptoms.

#### Temperament Evaluation of Memphis, Pisa, Paris, and San Diego Autoquestionnaire

The Temperament Evaluation of Memphis, Pisa, Paris, and San Diego Autoquestionnaire (TEMPS‐A) assesses affective temperaments.^14^ The validity and reliability of the TEMPS‐A,^18^ as well as that of the Japanese version,^19^ have been confirmed. The TEMPS‐A comprises the following five subscales: Cyclothymic Temperaments (21 items), Depressive Temperaments (21 items), Irritable Temperaments (20 items for men, 21 items for women), Hyperthymic Temperaments (21 items), and Anxious Temperaments (26 items). Subjects choose *true* (2 points) or *false* (1 point) for each question item. In this study, the final scores of each affective temperament were calculated by dividing the total scores of each of the subscales by the number of questions (i.e., mean value), according to the method of Matsumoto et al.^19^

#### Child Abuse and Trauma Scale

The Child Abuse and Trauma Scale (CATS) is a self‐administered questionnaire for assessing childhood abuse and trauma.^20^ The Japanese version has been reported to have good validity and reliability.^21^ CATS consists of 38 items, including sexual abuse (6 items), punishment (6 items), neglect/negative home atmosphere (14 items). Subjects rate each item by how frequently they experienced the particular maltreatment or abuse, using a 4‐point Likert scale. CATS total scores were used for the statistical analysis. Total scores are interpreted as proportional to the severity of childhood maltreatment and abuse.

### Statistical analysis

The associations of the subjects' demographic and clinical characteristics and questionnaire data with bipolar depressive severity were assessed using the *t*‐test and Pearson's correlation analysis.

The following hypotheses were tested using covariance structure analysis: (1) The latent variable “affective temperament” consists of observed variables of four TEMPS‐A subscales, excluding Hyperthymic Temperaments (i.e., Depressive Temperament, Cyclothymic Temperament, Anxious Temperament, and Irritable Temperament), (2) CATS total scores increase “affective temperament” scores, (3) “affective temperament” scores affect PHQ‐9 depressive scores, and (4) CATS total scores increase PHQ‐9 depressive scores indirectly, mediated by “affective temperament.” The above hypothetical model was assumed based on a previous study suggesting that affective temperaments mediate the association between neglect in childhood and the severity of major depression.^12^ As Rovai et al.^22^ reported that hyperthymic temperament differs from the other four temperaments (cyclothymic, depressive, irritable, and anxious temperaments) because only hyperthymic temperament may be protective against mood disorders, anxiety disorders, and substance use disorders, the four affective temperaments, excluding hyperthymic temperament, were included in the model. In the structural equation model, when the direct effect of CATS on PHQ‐9 is not significant but the indirect effect of CATS on PHQ‐9 via “affective temperament” is significant, “affective temperament” is regarded as a complete mediator between CATS and PHQ‐9. On the other hand, when CATS is significantly associated with PHQ‐9 both directly and indirectly through “affective temperament,” “affective temperament” is regarded as a partial mediator.^23^, ^24^

The goodness‐of‐fit of the model was tested using the following indices: χ², comparative‐fit index (CFI), Tucker–Lewis index (TLI), and the root‐mean‐square error of approximation (RMSEA). If χ² was not significant, this suggested that the difference between the predicted values and measured values was not significant, which was interpreted as a good fit. In addition, when CFI or TLI was more than 0.97, or the RMSEA was less than 0.05, the model was also interpreted as a good fit.^25^

Pearson's correlation analysis and the *t*‐test were conducted using SPSS Version 27 software (IBM, Armonk, NY, USA), and the covariance structure analysis was performed using Mplus Version 8.4 software (Muthén & Muthén, Los Angeles, CA, USA). The level of statistical significance was set at a *p*‐value of less than 0.05.

## RESULTS

### Associations of demographic and clinical characteristics of subjects with severity of depressive symptoms (PHQ‐9 scores)

The associations of demographic and clinical characteristics of subjects with the severity of depressive symptoms (PHQ‐9 scores) are shown in Table 1. Cyclothymic, depressive, irritable, and anxious temperaments and CATS scores significantly and positively correlated with PHQ‐9 scores, whereas age significantly and negatively correlated with PHQ‐9 scores. Additionally, PHQ‐9 scores were significantly higher in subjects without a family history of mood disorders in first‐degree relatives than those with a family history. However, no significant associations between melancholic features, sex, years of education, employment status, and hyperthymic temperament and PHQ‐9 scores were detected.

**Table 1:** Associations between PHQ‐9 scores and demographic characteristics.

Characteristic or measure	Value (number or mean ± SD)	Correlation with PHQ‐9 score
Sex (male:female)	42:33	Male: 8.0 ± 6.4 versus female: 8.5 ± 5.9, *p* = 0.71 (*t*‐test)
Age (years)	47.3 ± 11.6	*r* = –0.33, *p* = 0.004
Years of education	14.4 ± 2.2	*r* = –0.05, *p* = 0.67
Employment status (employed:unemployed)	41:34	Employed: 8.6 ± 5.7 versus unemployed: 7.4 ± 6.6, *p* = 0.32 (*t*‐test)
First‐degree relative with mood disorder (yes:no)	17:58	Yes: 5.4 ± 5.0 vs no: 9.0 ± 6.2, *p* = 0.03 (*t*‐test)
Melancholic features (yes:no)	26:49	Yes: 9.4 ± 6.8 vs no: 7.6 ± 5.7, *p* = 0.23 (*t*‐test)
PHQ‐9 score	8.2 ± 6.1	–
TEMPS‐A (average score)		
Depressive	1.50 ± 0.19	*r* = 0.55, *p* < 0.001
Cyclothymic	1.39 ± 0.28	*r* = 0.48, *p* < 0.001
Anxious	1.40 ± 0.25	*r* = 0.51, *p* < 0.001
Irritable	1.23 ± 0.21	*r* = 0.54, *p* < 0.001
Hyperthymic	1.24 ± 0.18	*r* = –0.03, *p* = 0.8
CATS total score	0.94 ± 0.66	*r* = 0.39, *p* < 0.001

*Note*: *r* = Pearson correlation coefficient.

Abbreviations: CATS, Child Abuse and Trauma Scale; PHQ‐9, Patient Health Questionnaire‐9; SD, standard deviation; TEMPS‐A, Temperament Evaluation of the Memphis, Pisa, Paris, and San Diego Autoquestionnaire.

### Covariance structure analysis

**Figure 1:**
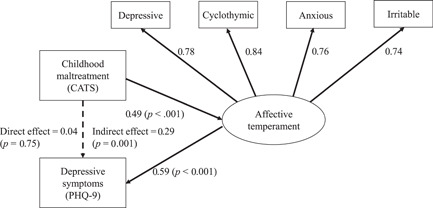
Structural equation model including childhood maltreatment, affective temperaments, and bipolar depression severity. Rectangles indicate observed variables, and the oval indicates the latent variable. Arrows with bold lines represent statistically significant paths, and the arrow with the broken line indicates a nonsignificant path. The numbers beside the arrows show the standardized path coefficients (minimum = −1, maximum = −1). CATS, Child Abuse and Trauma Scale; PHQ‐9, Patient Health Questionnaire‐9.

Figure 1 shows the results of the covariance structure analysis. The covariance structure analysis was controlled by the covariates of sex, age, and family history of mood disorders in first‐degree relatives. The model fit was good (χ² analysis, *p* = 0.32; RMSEA = 0.04, CFI = 0.99, and TLI = 0.98). The path coefficients from cyclothymic, depressive, irritable, and anxious temperaments to the latent variable of “affective temperament” were all positive and high, with cyclothymic temperament being the highest. The path coefficient from CATS scores to “affective temperament,” and that from “affective temperament” to PHQ‐9 scores were positive and significant, although the path coefficient from CATS scores to PHQ‐9 scores was not significant. The indirect effect of CATS scores on PHQ‐9 scores through “affective temperament” was significant (standardized indirect effect = 0.29; *p* = 0.001). The adjusted *R*² was 0.46 and significant (*p* < 0.001), indicating that 46% of the variance in PHQ‐9 scores can be explained by this model.

## DISCUSSION

In the present study, we showed for the first time the significant mediation effect of affective temperaments between childhood maltreatment and bipolar depression severity using covariance structure analysis. In other words, childhood maltreatment can increase affective temperaments, and thereby increase bipolar depression severity. The present finding is similar to our previous finding that childhood maltreatment can worsen depression severity via increasing affective temperaments in the general population and in people with major depression, and via changing personality traits of the Temperament and Character Inventory^26^ in people with schizophrenia.^10^, ^12^, ^27^ Taken together with the present results, these results suggest that the mediating effects of personality traits on the association between childhood maltreatment and depressive symptoms may occur independently of the presence or type of mental disorder.^10^, ^12^, ^27^ However, the direct effect from childhood maltreatment to bipolar depression severity was not significant, suggesting that the effect of affective temperaments mediating the impact of childhood maltreatment on bipolar depression was complete, and that affective temperaments were mainly involved in the influence of childhood maltreatment on bipolar depression severity.

In this study, childhood maltreatment significantly and positively predicted increases in affective temperaments, consisting of cyclothymic, depressive, irritable, and anxious temperaments in people with bipolar disorder. Our present findings are similar to those of our previous study that childhood maltreatment significantly positively predicted affective temperaments, consisting of cyclothymic, depressive, irritable, and anxious temperaments in a mixed group of healthy controls and people with bipolar disorder, by structural equation modeling.^13^ In addition, it has been reported that increases in cyclothymic, depressive, and anxious temperaments can be significantly predicted in people with bipolar disorder who experienced childhood trauma,^28^ which is similar to our present findings. Furthermore, in the present study, cyclothymic temperament had the highest path coefficient from the latent variable of “affective temperament” among the affective temperaments, which is interesting because cyclothymic temperament is known to be the most prevalent premorbid or characteristic affective temperament of bipolar disorder.^29^, ^30^, ^31^ In addition, our previous study demonstrated that childhood maltreatment increased affective temperaments in the general population and in people with major depression, and that anxious and cyclothymic temperaments were more strongly associated with childhood maltreatment than depressive and irritable temperaments in the general population. Furthermore, the anxious temperament was more powerfully associated with childhood maltreatment than depressive, cyclothymic, and irritable temperaments in people with major depression.^10^, ^12^ Therefore, childhood maltreatment might differently influence affective temperaments among the general population, people with major depression, and those with bipolar disorder.

The present study shows the association between childhood maltreatment and affective temperaments, and a mediating effect of affective temperaments on adulthood depression severity from childhood maltreatment in people with bipolar disorder. Therefore, in clinical practice, assessing the patient's history of childhood maltreatment and affective temperaments might help predict and understand the factors involved in the worsening of depressive symptoms in people with bipolar disorder.

### Limitations

This study has several limitations. First, the number of included subjects was small (75 subjects), and this may be the reason why the direct effect of childhood maltreatment on bipolar depression severity was insignificant. Second, it is difficult to conclude the causal effect of affective temperaments between childhood maltreatment and bipolar depression severity, because this study was a cross‐sectional study. To confirm the causal association, prospective longitudinal studies are required. Third, this study did not consider genetic factors that are considered to be important in the etiology of bipolar disorder. To elucidate the pathophysiological mechanism of bipolar depression, future studies using a more comprehensive model, including genetic factors, are needed.

### Conclusion

This study showed that childhood maltreatment can increase affective temperaments, which can in turn aggravate depressive symptoms in people with bipolar disorder. To our knowledge, this is the first study to identify the association between childhood maltreatment and negative affective disorder in people with bipolar disorder and the mediation effect of affective temperaments between childhood maltreatment and bipolar depression severity using covariance structure analysis. The present findings have the potential to help elucidate the mechanism of bipolar depression. More extensive prospective cohort studies that also consider genetic factors will be necessary in the future for further elucidation of the etiology of bipolar depression.

## AUTHOR CONTRIBUTIONS

All authors made substantial contributions to the conception and design of the study, acquisition of the data, or analysis and interpretation of the data; took part in drafting the manuscript or revising it critically for important intellectual content; gave final approval of the version to be published; and agree to be accountable for all aspects of the work.

## CONFLICTS OF INTEREST STATEMENT

Jiro Masuya has received personal compensation from Otsuka Pharmaceutical, Eli Lilly, Astellas, and Meiji Yasuda Mental Health Foundation, and grants from Pfizer. Yota Fujimura has received research and grant support from Novartis Pharma, Otsuka Pharmaceutical, Astellas, Dainippon Sumitomo Pharma, and Shionogi. Shinji Higashi has received honoraria from Dainippon Sumitomo Pharma and Novartis Pharma. Takeshi Inoue has received personal compensation from Mochida Pharmaceutical, Takeda Pharmaceutical, Eli Lilly, Janssen Pharmaceutical, MSD, Taisho Toyama Pharmaceutical, Yoshitomiyakuhin, and Daiichi Sankyo; grants from Shionogi, Astellas, Tsumura, and Eisai; and grants and personal compensation from Otsuka Pharmaceutical, Dainippon Sumitomo Pharma, Mitsubishi Tanabe Pharma, Kyowa Pharmaceutical Industry, Pfizer, Novartis Pharma, and Meiji Seika Pharma; and is a member of the advisory boards of Pfizer, Novartis Pharma, and Mitsubishi Tanabe Pharma. Ichiro Kusumi has received personal compensation from Astellas, Chugai Pharmaceutical, Daiichi Sankyo, Dainippon Sumitomo Pharma, Eisai, Eli Lilly, Janssen Pharmaceutical, Kyowa Hakko Kirin, Meiji Seika Pharma, MSD, Nippon Chemiphar, Novartis Pharma, Ono Pharmaceutical, Otsuka Pharmaceutical, Pfizer, Mitsubishi Tanabe Pharma, Shionogi, and Yoshitomiyakuhin; has received research/grant support from AbbVie GK, Asahi Kasei Pharma, Astellas, Boehringer Ingelheim, Chugai Pharmaceutical, Daiichi Sankyo, Dainippon Sumitomo Pharma, Eisai, Eli Lilly, GlaxoSmithKline, Kyowa Hakko Kirin, Meiji Seika Pharma, MSD, Novartis Pharma, Ono Pharmaceutical, Otsuka Pharmaceutical, Pfizer, Takeda Pharmaceutical, Tanabe Mitsubishi Pharma, Shionogi, and Yoshitomiyakuhin; and is a member of the advisory boards of Dainippon Sumitomo Pharma and Tanabe Mitsubishi Pharma. The other authors declare that they have no actual or potential conflicts of interest associated with this study. Yu Tamada received honoraria from Otsuka Pharmaceutical, Dainippon Sumitomo Pharma, and Eisai.

## ETHICS APPROVAL STATEMENT

This study was approved by the Institutional Review Boards of Tokyo Medical University, National Defense Medical College, and Hokkaido University Hospital (research approval number SH4098) and was conducted in accordance with the Declaration of Helsinki, as revised in 2008.

## PATIENT CONSENT STATEMENT

Written consent was obtained from all the subjects at study entry.

## CLINICAL TRIAL REGISTRATION

N/A.

## Data Availability

Data cannot be shared publicly because of Ethics Committee restriction. All relevant data are within the paper. Data are available from the Internal Review Board of the Department of Psychiatry, Tokyo Medical University (Japan) (contact via email: seisinka@tokyo-med.ac.jp) for researchers who meet the criteria for access to confidential data.
